# Film Excerpts Shown to Specifically Elicit Various Affects Lead to Overlapping Activation Foci in a Large Set of Symmetrical Brain Regions in Males

**DOI:** 10.1371/journal.pone.0022343

**Published:** 2011-07-27

**Authors:** Sherif Karama, Jorge Armony, Mario Beauregard

**Affiliations:** 1 McConnell Brain Imaging Center, Montreal Neurological Institute, McGill University, Montreal, Canada; 2 Douglas Mental Health University Institute, Department of Neurology and Neurosurgery, McGill University, Montréal, Quebec, Canada; 3 Centre de Recherche en Sciences Neurologiques, Université de Montréal, Montréal, Québec, Canada; Kyushu University, Japan

## Abstract

While the limbic system theory continues to be part of common scientific parlance, its validity has been questioned on multiple grounds. Nonetheless, the issue of whether or not there exists a set of brain areas preferentially dedicated to emotional processing remains central within affective neuroscience. Recently, a widespread neural reference space for emotion which includes limbic as well as other regions was characterized in a large meta-analysis. As methodologically heterogeneous studies go into such meta-analyses, showing in an individual study in which all parameters are kept constant, the involvement of overlapping areas for various emotion conditions in keeping with the neural reference space for emotion, would serve as valuable confirmatory evidence. Here, using *f*MRI, 20 young adult men were scanned while viewing validated neutral and effective emotion-eliciting short film excerpts shown to quickly and specifically elicit disgust, amusement, or sexual arousal. Each emotion-specific run included, in random order, multiple neutral and emotion condition blocks. A stringent conjunction analysis revealed a large overlap across emotion conditions that fit remarkably well with the neural reference space for emotion. This overlap included symmetrical bilateral activation of the medial prefrontal cortex, the anterior cingulate, the temporo-occipital junction, the basal ganglia, the brainstem, the amygdala, the hippocampus, the thalamus, the subthalamic nucleus, the posterior hypothalamus, the cerebellum, as well as the frontal operculum extending towards the anterior insula. This study clearly confirms for the visual modality, that processing emotional stimuli leads to widespread increases in activation that cluster within relatively confined areas, regardless of valence.

## Introduction

The limbic system theory is arguably the most famous view on the neural architecture of human emotion [1]. According to this theory, all aspects of emotions are mediated by a specialized group of brain structures forming an integrated neural system [2], [3]. Although this single-system view of emotions continues to be part of common medical and scientific parlance, its validity has been questioned on multiple grounds ranging from neuroanatomy and neuropathology to brain imaging [2], [4], [5]. This being said, a broad single-system view of emotions remains attractive to many despite there being no clear consensus as to what exact regions may constitute it [2], [6], [7]. A single system for processing emotions would obviously be made up of subsystems relatively specialized in different aspects of emotions. However, if such a system indeed exists, a broad description of the set of areas that constitute it could serve as an important step to more meaningful analyses of the respective contributions and dynamics of its subsystems [8].

In the last few years, meta-analyses have begun to tackle such issues by examining patterns of activation across brain imaging studies of emotion (emotion is used throughout the text to refer to both so-called discrete emotions [e.g. disgust] as well as to affective states [e.g. sexual arousal]) [2], [9], [10], [11]. While all of these meta-analyses have sought to identify patterns of activation specific to given emotional states, some have also examined findings across imaging studies “in search of specific regions associated with emotional activation in general” [9]. Although these meta-analyses have provided important insights through improved statistical power over single studies, their assessment of the degree of overlap across emotions may still have been influenced by methodological issues such as the relying on reported peaks of activation within broader areas of activation [12].

Recently, a neural reference space for emotion-related phenomena (defined as “the set of brain regions thought to instantiate emotions and related affective states” [8]) was characterized in a large meta-analysis using an analytical approach relatively less susceptible to reporting conventions between individual studies than approaches used by other similar meta-analyses [8]. While the neural reference space for emotion and the network of areas that characterizes the limbic system do share some common areas, they are not isomorphic constructs. According to this meta-analysis, the neural reference space for emotion was observed not only to comprise many areas within the original limbic system but to further consist of areas not traditionally part of the limbic system such as the frontal operculum, the superior temporal cortex, the basal ganglia, the insula, the brainstem, and the cerebellum [8].

While individual studies of emotion have generally reported the involvement of limbic and paralimbic structures, few have explicitly commented on the overlap across emotion conditions [10], [13], [14], [15], [16], [17], [18], [19], [20], [21], [22], [23], [24], [25]. Those that have looked at the overlap have generally reported a relatively modest across-emotion overlap when compared with the widespread regions constituted by the neural reference space for emotion. While this may be due to power issues as alluded to by Phan et al. (2002), it may also in part be due to the fact that studies that have looked at the overlap have done so using visual inspection alone instead of a formal conjunction analysis. On the other hand, as meta-analyses compound methodologically heterogeneous studies, it remains possible that the joint contribution of different effects from various studies led to the large number of foci defining the neural reference space for emotion.

Because of such issues, being able to show in an individual study, in which all parameters are kept constant, the involvement of overlapping areas for various emotion conditions in keeping with the neural reference space for emotion would serve as important complementary and confirmatory evidence. Here, such an attempt was made using functional magnetic resonance imaging (*f*MRI) to formally examine, through a stringent conjunction analysis, the extent of overlap in regions exhibiting changes in activation during viewing of validated effective emotion-eliciting film excerpts known to quickly and specifically elicit disgust, amusement, or sexual arousal (note that other emotions like fear, sadness, or anger, for instance, can be difficult to consistently and quickly elicit out of context and usually require longer exposure times to eliciting stimuli [26]). The decision of using film excerpts was not only made on the basis of their being more naturalistic than static images but also because they have been shown to be among the most effective means of eliciting specific target emotions [26], [27], [28], [29].

What distinguishes the current work from previous studies on emotion is that it uses, in combination, many strategies to improve sensitivity so as to maximise the chances of confirming an overlap between emotions if it exists. While each of these strategies has been used in previous efforts, this is the first time that they are used in combination. These strategies include: 1) using validated film excerpts instead of still pictures; 2) using a formal conjunction analysis instead of visual inspection to assess overlap; 3) using a sensitive short epoch box-car design (see methods for rationale) instead of an event-related protocol. Note that most recent studies have used an event-related protocol instead of a box-car design. While the use of event-related protocols can be preferable and justifiable in many cases, it remains, all being equal, less powerful/sensitive than a box-car design [30]).

It should be emphasized that the goal of the current work is not to disambiguate the specific functions (*e.g.*, cognitive processing and appraisal, adaptive physiological changes, motivation, preparation to act…) of the various components of a putative general emotion system or network but to confirm its existence and identify its constituting elements for the visual modality.

For the current work, regions of interest (ROIs) for studying the degree of overlap across emotions were all the areas of the neural reference space identified previously by Wager et al. (2008). These comprised localized foci within the periaqueductal gray, thalamus, subthalamic nucleus, hypothalamus, amygdala, hippocampus, basal ganglia, insula, frontal operculum, anterior and posterior cingulate gyrus, temporal pole, medial prefrontal cortex, inferior frontal cortex, pre-supplementary motor area (SMA), posterior inferior temporal cortex, posterior superior temporal cortex, striate cortex, lateral occipital cortex, temporo-occipital junction, and cerebellar hemispheres.

A secondary (and exploratory) aim of the current work was also to locate foci of activation specific to each of the three target emotions. Regions that were expected to exhibit significantly higher levels of activation for a given emotion compared with the other two emotions were difficult to determine given the few studies directly contrasting the emotions examined here. In light of this and given that this aim was secondary and exploratory, no ROIs were *a priori* determined for these analyses.

## Methods

The ethics committee of the Research Center of the University of Montreal Hospital Center (Notre-Dame Hospital) approved the study. Subjects gave written informed consent to participate in this study.

### Subjects

In order to avoid any potential confounding effect of hormonal changes associated with the menstrual cycle this study was exclusively conducted on 20 right-handed male subjects. The age of these subjects ranged from 21 to 30 years (mean ± sd: 25.5±3.4). Handedness was determined by means of the Edinburgh Laterality Scale [31]. Exclusion criteria included a history of neurological or psychiatric illness. All subjects gave informed written consent after the nature of the experiment was explained.

### Selection and validation of stimuli

Two types of silent visual stimuli were used: neutral film excerpts and emotion-eliciting film excerpts.

#### Matching of Neutral and Emotion-eliciting stimuli

In order to minimize confounders, excerpts from both neutral and emotion conditions were matched as closely as possible. Both types of stimuli included exterior and interior scenes as well as daytime and evening scenes. Further, both sets of stimuli were in color and depicted scenes of social interaction. There were no systematic image intensity differences between the two sets of stimuli.

#### Neutral Stimuli

Stimuli designated as neutral were chosen from a series of more than 120 short film excerpts selected by the present investigators on the basis of their believed lack of potential to induce any significant emotional reaction. These excerpts, which were extracted from movies or documentaries, depicted various scenes of social interactions (e.g. gardening, renovation, etc…). Ten male subjects (mean age ± sd: 26±2) who did not participate in the *f*MRI aspect of the study were recruited for the validation of these excerpts. Each subject, sitting alone in a room, had to report on scales, each ranging from 0 (lowest) to 8 (highest), the levels of sexual arousal, surprise, amusement (specified to be impulse to laugh), sadness, fear, disgust, and anger produced by each excerpt. For each excerpt, an average score was calculated as a compound of sexual arousal and of the six basic emotions [32]. Thirteen excerpts had an average score below 1 for each emotion and were collated in order to make up nine 33-sec blocks of neutral stimuli.

#### Emotion-eliciting stimuli

The emotion-eliciting film excerpts depicted scenes shown to elicit amusement (comedy), disgust (scenes of mutilation), or sexual arousal (explicit male-female interactions). These stimuli, which can be viewed as generally activating in nature (i.e., leading to increased vigilance or arousal), were selected given their potential to quickly (i.e., within 30 sec) induce an emotional state.

For each 33-sec excerpts, the same validation procedure as that used for the neutral stimuli was implemented with the same ten male subjects. In order to minimize surprise, subjects were told that they would be viewing emotionally-laden film excerpts aimed at eliciting amusement, disgust, or sexual arousal.

For the amusement-eliciting stimuli, the three excerpts with the highest reported average levels of amusement were retained from a series of 21 such excerpts. These three excerpts had, respectively, average scores of 4.1 (SD = 1.4), 4.3 (SD = 1.5), and 4.4 (SD = 1.7) for amusement, and 1.5 or below for each of the other emotions.

For the disgust-eliciting stimuli, the three excerpts with the highest reported average levels of disgust were retained from a series of 29 such excerpts. These three excerpts had, respectively, an average score of 5.3 (SD = 1.6), 6.0 (SD = 1.3), and 4.9 (SD = 1.5) for disgust and 1.1 or below for each of the other emotions.

For the sexual arousal-eliciting stimuli, the three excerpts with the highest reported average levels of sexual arousal were retained from a series of 20 such excerpts. These three excerpts had a mean score of 4.4 (SD = 1.3), 4.5 (SD = 1.4), and 4.7 (SD = 1.2) for sexual arousal and below 1 for each of the other emotions.

### Experimental design

The reason for selecting stimuli that could quickly induce an emotional state was to minimize, as much as possible, known low frequency noise artefacts inherent to long epoch *f*MRI designs. The vast majority of studies that have used a box-car design in combination with film excerpts to study emotions have done so with relatively long condition durations (typically more than 90 sec/block). Such a methodological strategy has been justified on grounds that a long duration of a given emotion condition is needed to elicit a strong enough emotion and corollary BOLD signal changes in the brain. However, this strategy ignores the possibility that technical limitations inherent in fMRI (e.g. low frequency noise is known to have a potentially significant impact with condition length above 40 sec [33], [34], [35]) may actually more than outweigh its purported benefits.

BOLD signal changes were measured during each of the three experimental conditions: amusement, disgust, and sexual arousal. There was one run per condition and each subject was exposed to the three runs in random order. There was a 5 min break between runs to make sure subjects were still comfortable and to ask them for their ratings after each run (see below). In each run, subjects were exposed to both three neutral and three condition specific 33-sec blocks. Order of presentation of the neutral and respective emotional condition blocks within a run was randomized and varied between subjects. Further, the order of presentation of the various runs was randomised between subjects. Neutral and condition-specific blocks were interleaved with 33-sec baseline blocks during which subjects viewed a blank cyan screen. Each run began with such a baseline block. It is important to note that having nine neutral blocks allowed for the use of different neutral stimuli for each emotional condition (i.e., three neutral blocks per condition). Finally, the order of presentation of the various runs was randomised between subjects.

Prior to scanning (which was mainly done between 1pm and 5pm), subjects were told to simply relax and watch the film clips. To minimize surprise, they were also told that they would be viewing short emotionally-laden film excerpts aimed at inducing amusement, disgust, or sexual arousal. After each run, subjects were asked to rate, from 0 to 8, the average levels of sexual arousal, surprise, amusement, sadness, fear, disgust, and anger experienced during the run.

### Stimuli presentation and scanning parameters

During *f*MRI sessions, film clips were presented through goggles connected to an MR compatible video system (Resonance Technology, Inc., Van Nuys, CA, USA). MR compatible earphones were provided but were used only to convey instructions to the subjects. Sound was turned off during the runs. Earplugs were used to minimize the distracting sound of the fMRI device. Echoplanar images (EPI) were acquired on a 1.5-Tesla system (Magnetom Vision, Siemens Electric, Erlangen, Germany). Twenty-eight slices (5 mm thick) were acquired in an inclined axial plane, aligned with the AC-PC axis. T2*-weighed functional images were acquired using an EPI pulse sequence (TR = 3000 msec, TE = 54 msec, Flip = 90°, FOV = 215 mm, Matrix = 64×64, Voxel size ∼3.4 mm^2^). Following functional scanning, high-resolution data were acquired via a T1-weighted three-dimensional volume acquisition obtained using a gradient echo pulse sequence (TR = 9.7 msec, TE = 4 msec, Flip = 12°, FOV = 250 mm, Matrix = 256×256, Voxel size ∼1 mm^2^). One hundred thirty-two volumes were acquired in each run and each subject underwent three sessions for a total of 396 volumes acquired per subject.

### Image pre-processing

The first three volumes of each run (which were within the baseline condition – i.e. while seeing a blank cyan screen) were discarded to minimize artifacts that are known to occur in the first few volumes. Every functional brain volume of each subject was then visually examined to identify artifacts due to head movement or to scanner field distortions. Head movement was corrected using a 6 parameter (rigid body) spatial transformation with the fourth image (i.e., after discarding the first three) serving as the reference image. Plots of the transformation were also examined to assess degree of movement for each subject for each run. No epoch-correlated head movements were noted. However, one subject had to be eliminated from the analysis due to too much (>1.5 mm in x, y, or z axis) movement during two of the three runs and another subject had to be eliminated due to a malfunction of the goggles. This left 18 subjects for statistical analyses.

### Image analysis

Data were analyzed using Statistical Parametric Mapping (SPM5, Wellcome Department of Cognitive Neurology, London, UK). Scans were spatially normalized using the standard Montreal Neurological Institute (MNI) template. Images were then convolved in space with a three-dimensional isotropic gaussian kernel (full width at half maximum –FWHM- of 8 mm) to improve the signal-to-noise ratio and to accommodate for residual variations in functional neuroanatomy that usually persist between subjects after spatial normalization. High pass filtering was set to 264 sec. as warranted by examination of the domain frequency plots. For each condition, effects at every voxel were estimated using the general linear model. For each run, the emotional and neutral conditions were explicitly modeled in the design matrix while the baseline condition was modeled implicitly. First-level ‘Amusement minus Neutral’, ‘Disgust minus Neutral’, and ‘Erotica minus Neutral’ were produced for each subject. A second-level, ‘mixed-effects’ model with the subjects as the random-effect factor was implemented to produce the ‘Amusement minus Neutral’, ‘Disgust minus Neutral’, and ‘Erotica minus Neutral’ contrasts for the whole group. Corrections for non-sphericity were applied. Voxel values yielded a statistical parametric map of the *t* statistic. Height threshold was set to p≤0.001, uncorrected for multiple comparisons, for the *a priori* determined regions of interest and only clusters comprising at least 10 voxels were examined. In order to be considered inside a region of interest, peak activation foci had to be within 10 mm, in either hemisphere, of regions identified by Wager et al. (2008) and be inside the same anatomical structure as these peak coordinates. Foci that survived the 0.001 threshold but that were not *a priori* determined are reported for convenience only and should be viewed as trends unless they survived a 0.05 FWE-corrected threshold. Indeed, using an uncorrected p = 0.001 threshold is very likely to produce false positives given the number of comparisons. However, when one finds positive signal that has frequently been reported in prior studies, the chance that this signal is a false positive is greatly decreased. The reverse is also true: the chance that a signal is a false positive becomes relatively high when few or no previous studies have reported the finding.

As visual inspection of functional images or of tables of peaks may make it difficult to unveil subtle yet genuine overlaps between emotional conditions, a conjunction analysis of the three second-level contrasts [36], [37], [38] was implemented. As we explicitly wanted to assess overlap in regions independently activated in each emotional condition, the very conservative conjunction null method described by Nichols et al. (2005) was used. The conjunction null method is more conservative than the conjunction global method. For a voxel to be identified as activated using the conjunction null method, it has to be independently activated in each of the examined comparisons. In contrast, for a conjunction analysis using the global null method, a voxel can be considered activated due to the ‘sum’ of effects of the examined comparisons. In other words, the global null method may lead to the categorization of a voxel as significantly activated even if it's not activated in all contrasts (e.g. in situations where a highly significant activation in some of the contrasts statistically ‘outweighs’ the non-significant contrasts). Similarly, it can also lead to the categorization of a voxel as significantly activated in situations where no contrast by itself yields a significant activation of the given voxel but in which the combination/sum of contrasts yields it as significant. For more details regarding the differences between the conjunction null and global null methods, see Nichols et al. (2005) and Friston et al. (2005).

Regions of interest were determined as above. The threshold for the conjunction null analysis was also set to an uncorrected p value of 0.001 for these regions of interest. As the neutral stimuli differed between contrasts, this ended up being relatively strict for a conjunction as a given voxel had to survive a 0.001 threshold for each and every contrast (i.e. amusement>neutral, disgust>neutral, and sexual arousal>neutral). Here also, regions that survived the 0.001 threshold but that were not *a priori* determined are reported for convenience only and should be viewed only as mild trends unless they survived a 0.05 FWE-corrected threshold.

In order to examine activation foci specific to each emotion condition, each emotion was contrasted against the union of the other two emotions after weighing each of the latter by a factor of 0.5. For instance, to identify foci specific to amusement, the following contrast was used: (Amusement>Neutral)>0.5*[(Disgust>Neutral)+(Sexual Arousal>Neutral)]. The same process was implemented for Disgust and Sexual Arousal. The same threshold scheme as the one implemented above was also used here.

## Results

From a subjective point of view, the film excerpts within the ‘disgust condition’ run were reported to induce transient states of disgust (mean ± SD: 5.0±1.5). Similarly, transient states of amusement were reported for the ‘amusement condition’ run (3.6±1.5) and transient states of sexual arousal for the ‘sexual arousal condition’ run (4.6±1.2). For each run, reported mean transient emotional states for other emotions than the target emotion were all below 1.5.

**Figure 1:**
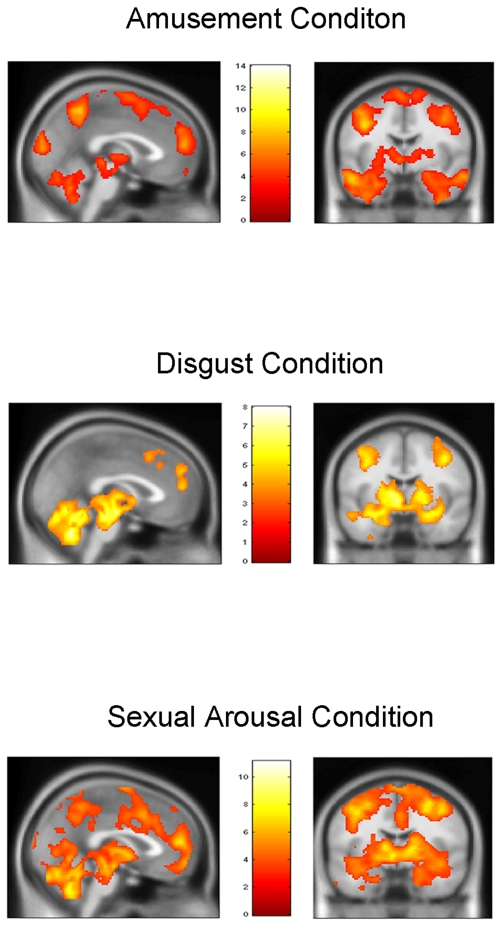
Sagittal and coronal slices across the main areas of supra-threshold areas of activation for the ‘Amusement condition minus Neutral’, ‘Disgust condition minus Neutral’, and ‘Sexual arousal condition minus Neutral’ contrasts. Note the similarities between contrasts. All contrasts clearly show involvement of medial prefrontal cortex, supplementary motor area, hypothalamus, thalamus, amygdala, ventral insula, and cerebellum.

**Figure 2:**
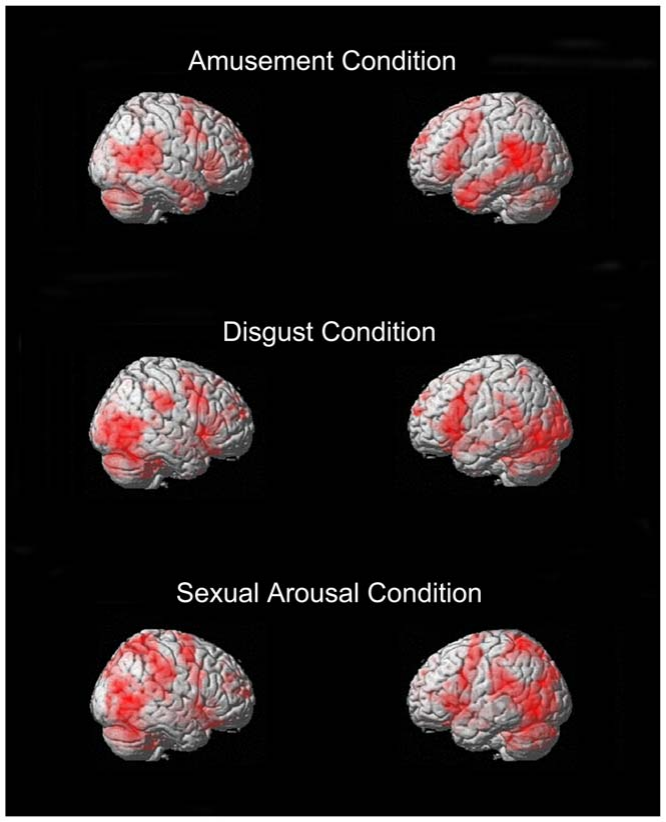
Three-dimensional rendered images of areas of supra-threshold areas of activation for the same contrasts as Figure 1. Here again, there similarities are obvious between contrasts. All contrasts clearly show involvement of the frontal operculum, dorsolateral prefrontal cortex, premotor and motor cortices, temporo-occipital regions, and cerebellum.

The Amusement minus Neutral, Disgust minus Neutral, and Sexual Arousal minus Neutral contrasts each revealed symmetrical, bilateral, and widely distributed foci of activation in *a priori* determined ROIs that included the medial prefrontal cortex, the anterior cingulate, the temporo-occipital junction, the basal ganglia, the brainstem, the amygdala, the hippocampus, the thalamus, the subthalamic nucleus, the posterior hypothalamus, the cerebellum, as well as the inferior frontal gyrus at the level of the frontal operculum extending towards the anterior insula. This was the case despite the fact that different neutral film excerpts were used for each of the respective neutral conditions. For a more exhaustive list of the various foci of activation, see Figures 1 and 2 as well as supporting information Tables S1, S2, and S3).

**Figure 3:**
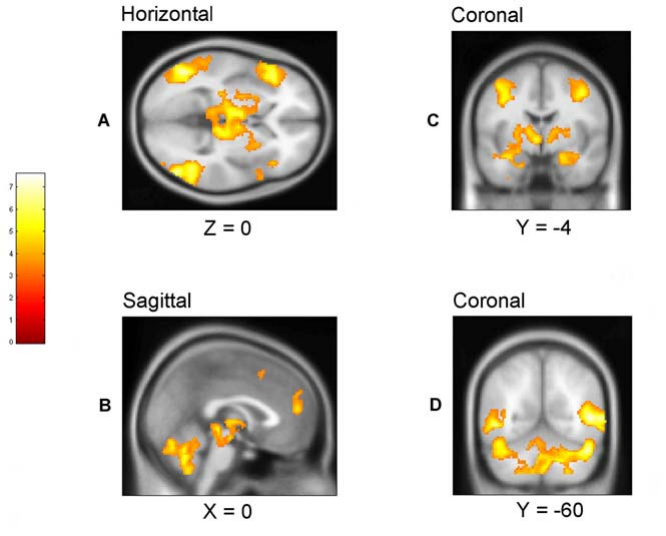
Areas of significant BOLD signal changes for the ‘amusement minus neutral’, ‘disgust minus neutral’, and ‘sexual arousal minus neutral’ conjunction null analysis conducted at the second level. Accompanying sagittal, coronal and horizontal slices through most ROIs are provided. BOLD signal changes for these slices are coregistered on top of normalized brain sections from the Montreal Neurological Institute (MNI) stereotaxic space template. Coordinates (x, y, and z) are given in mm and refer to locations in MNI stereotaxic space. Height threshold is set to p = 0.001, uncorrected. The neurological convention has been chosen for orientation of coronal sections in all figures i.e., left is left and right is right. **A:** Medial prefrontal cortex, frontal operculum encroaching on anterior insula, thalamus, lateral occipital gyrus **B:** Medial prefrontal cortex, anterior cingulate, thalamus, brainstem, cerebellum **C:** Supplementary motor area, thalamus, amygdala **D:** Temporo-occipital junction, cerebellum.

**Figure 4:**
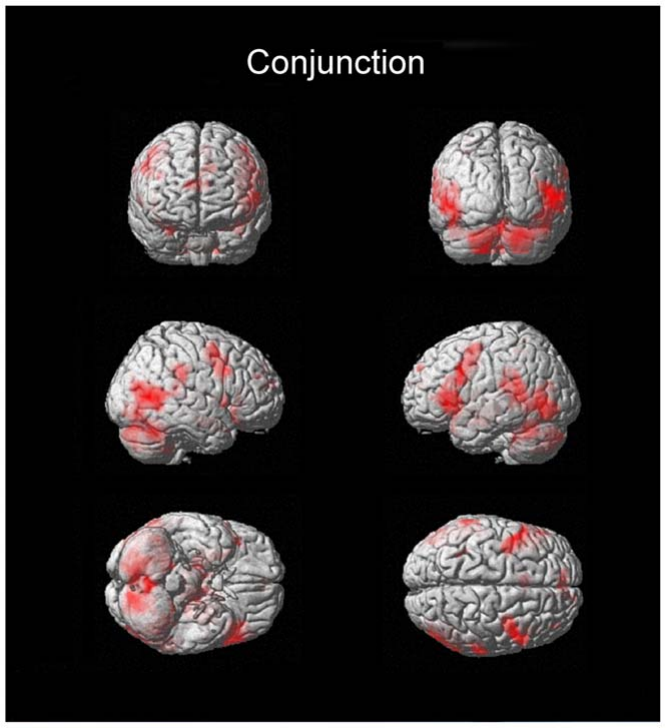
Three-dimensional rendered images of areas of significant activation for the same contrast as Figure 3. Note the involvement of the frontal operculum, temporo-occipital regions, cerebellum, and lateral temporal, premotor as well as motor cortices.

The conjunction null analysis formally confirmed the substantial overlap between emotional conditions of each of the above mentioned brain regions (see Figures 3 and 4 as well as supporting information Table S4).

**Figure 5:**
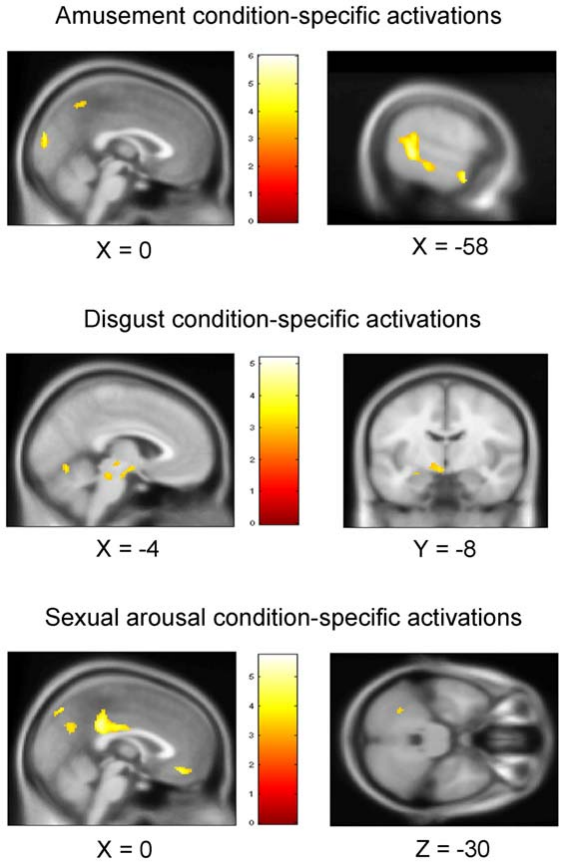
Sagittal, coronal, and/or axial slices across the main areas of supra-threshold activated areas for the ‘Amusement condition-specific’, ‘Disgust condition-specific’, and ‘Sexual arousal condition-specific’ contrasts. Slices were selected so as to show most involved areas. Amusement-specific temporal, medial occipital and precuneus areas are shown on top. Disgust condition-specific periaqueductal, posterior pons, left amygdala, left hypothalamus, and cerebellar areas are shown in the middle and Sexual arousal-condition-specific medial prefrontal, posterior cingulate, cerebellar, and precuneus regions are shown at the bottom.

**Figure 6:**
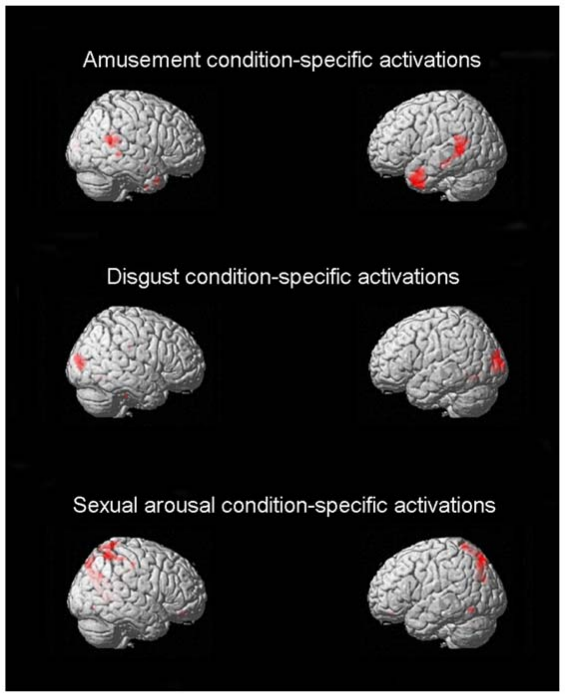
Three-dimensional rendered images of supra-threshold of activated areas for the same contrasts as Figure 5. Temporal cortical involvement is shown to be specific here to the Amusement condition, lateral occipital and fusiform areas to the Disgust condition, and superior parietal and posterior inferior temporal areas to the Sexual arousal condition.

Activation foci preferentially activated by the amusement condition, when contrasted with the disgust and sexual arousal conditions, comprised the *a priori* determined temporal cortex regions and bilateral temporo-parieto-occipital cortex (see Figures 5 and 6 as well as supporting information Table S5). Similarly, the *a priori* determined insula, frontal operculum, globus pallidus, amygdala, fusiform and lateral occipital areas were all shown to be preferentially activated by the disgust condition when contrasted with the amusement and sexual arousal conditions (see Figures 5 and 6 as well as supporting information Table S6). For the sexual arousal condition, the *a priori* determined ventral striatum, amygdala, anterior cingulate cortex, ventral medial prefrontal cortex, and precuneus were shown to be preferentially activated when contrasted with the amusement and disgust conditions (see Figures 5 and 6 as well as supporting information Table S7).

## Discussion

The most striking finding of the current work is arguably the clear across-emotion overlap of activation foci that includes various limbic and paralimbic areas. Finding this overlap for such a wide range of validated emotional stimuli strongly suggests the existence of a large network preferentially involved in processing emotional visual stimuli that are activating in nature, regardless of their valence. Importantly, results fit remarkably well with the neural reference space for emotion as characterized by Wager et al. (2008). Indeed, of all the structures identified by Wager et al. (2008), essentially only the cuneus and pre-SMA failed to survive the conjunction analysis. Conversely, foci within the SMA, superior posterior temporal cortex, inferior parietal lobule, inferior occipital cortex, temporo-occipital junction, posterior cingulate gyrus, and vermis were observed here despite not having been among the regions of the neural reference space characterized by Wager et al. (2008). Of these, foci within the SMA, the inferior parietal lobule, the inferior occipital cortex, and the temporo-occipital junction survived the very stringent 0.05 FWE-corrected threshold for the conjunction analysis, suggesting that they are genuinely involved regions despite not having been determined *a priori*.

While the goal of the current work was mainly to confirm the existence and describe the constituting elements of a neural reference space for emotion for the visual modality, nothing precludes speculations about the observed pattern of activation. Along those lines and in light of the nature of the selected stimuli, it could be speculated that activation in some of the observed areas may be partially modulated by the greater attention paid to emotion-inducing stimuli as a consequence of their being intrinsically more ‘interesting’ than their neutral counterparts. This would certainly be compatible with a suggested role in selective attention for the anterior insula [39] and the known modulation of extrastriate areas by selective attention [40]. Whatever may be the case, it should be stated that the modulation of attention for the processing of emotional stimuli is likely unavoidable. In fact, it could be argued that while not all stimuli that draw our attention are emotionally laden (e.g. salient objects), stimuli that do not draw our attention are likely not those that are consciously interpreted as highly emotional. Having said this, it would be rather implausible that all areas of the emotion network observed here were solely activated due to selective attention differences between neutral and emotional conditions as most regions have not generally been associated with attention modulation [41], [42].

As some of the task-positive network regions are involved here, it could be argued that the network observed here is, in fact, the task-positive network [43], [44], [45]. The task-positive network comprises a set of regions that are routinely activated together and believed to be involved in focused attention and working memory [43]. These regions are typically found to be anticorrelated with another set of regions which are routinely deactivated by the same tasks that lead to activation of the task-positive network [43]. Finding areas in common with the task-positive network is not surprising as, has been argued above, emotional stimuli likely do command more attention than neutral stimuli. However, ascribing the pattern of activation simply to the task-positive network is a position that is difficult to defend given that 1) the network observed here goes well beyond the task-positive network, involving areas of the cerebellum, many subcortical structures, as well as the medial prefrontal cortex, that are not part of the task-positive network and that 2) typically accompanying deactivated regions (i.e. the task-negative network) were absent. In fact, some of the regions expected to be found ‘de-activated’ (as part of the task-negative network) when eliciting the task-positive network were actually found to be activated (e.g. medial prefrontal cortex).

Evidence of brainstem involvement is a notable feature of the set of regions found activated here as it has frequently been identified as important in animal models of affective behaviour [46]. Nuclei within the brainstem have reciprocal connections with the medial prefrontal cortex, the insula, and the amygdala, among others [47], [48], [49]. In keeping with this, these three regions were all shown here to be activated across emotional conditions. Finding brainstem activation in the midbrain periaqueductal gray (PAG) is compatible with its known role in modulating various homeostatic and other physiological reactions including heart rate, blood flow to the face, and pupillary dilation [50]; reactions that may all be part of a coordinated emotional response.

The frontal operculum, observed here at a level contiguous with the anterior insula, is one of the areas most consistently reported as activated across brain imaging studies [51]. This region has been hypothesized to serve a critical role in conceptual processing associated with meaning such as that found in appraisal [8]. In line with this, manipulation of the context in which painful or emotion-inducing stimuli are presented modulates activation in this area [52].

Given the lack of a motor component to the current study, SMA activation may appear surprising. Yet, accruing evidence supports the existence of anatomo-functional links between traditional limbic areas and premotor/motor areas via cingulate and prefrontal cortical areas [53]. In fact, it has been shown that emotional states generated by visual cues can trigger movements through SMA involvement while movements in response to neutral visual cues do not elicit preferential SMA activation [53]. Such evidence supports the view that the SMA may be part of a neural network by which emotions interact with and modify motor planning [53].

Similarly, as the cerebellum has traditionally been mostly viewed as playing a crucial role in coordination and motor control, it is tempting to speculate that its involvement here may be related to motor planning during affective states. While this may be the case, evidence has begun to also suggest a more direct role in emotion-related phenomena. Indeed, it has been shown that cerebellar lesions can produce a blunting of affect and inappropriate social conduct [54] while stimulation of the cerebellar vermis may induce a pattern of behavior suggestive of arousal in animals [55]. Importantly, the cerebellum is connected with a network of ‘traditional’ limbic structures such as the hypothalamus, the dorsomedial prefrontal cortex, and the dorsomedial thalamic nucleus [8].

It is noteworthy that the posterior inferior frontal gyrus, the adjacent ventral premotor cortex, the inferior parietal lobule, as well as the posterior superior temporal sulcal area were all observed here as activated across emotion conditions. While the first three regions have been reported to exhibit mirror neuron properties and to constitute the human mirror neuron system (MNS), the fourth is believed to provide visual input to this system [56]. Together, all four regions make-up neural circuitry viewed as crucial for imitation which, in turn, is considered central for the development of fundamental social skills as it is believed, among other things, to be involved in understanding the goals, intentions, and desires of others [57]. As noted in the last few years, the parietal MNS appears to be involved in the motoric description of an action [58], [59] while the frontal MNS appears more concerned with the goal of the action [58], [60]. As it could be argued that assessment of intentions in emotional situations is generally more useful and perhaps more computationally demanding than in so-called ‘neutral’ situations, it could be speculated that finding activation of this network in the current context may be related to its relative sensitivity to socio-emotional cues.

For extensive discussions about the potential roles in emotion of other areas observed here, see Phan et al, (2002), Murphy et al. (2003), Wager et al., (2003, 2008) and Kober et al. (2008). Overall, as the network observed here includes brain regions known, from previous work, to be intimately involved in homeostasis, arousal, appraisal, and attention, results could be speculated to suggest the existence of a set of areas meant to improve the way we deal with activating emotional stimuli as these are arguably the ones with the greatest potential of having an immediate impact on our lives.

The current work also provides results pertaining to regions preferentially activated for each of the three emotion conditions studied here. All three emotion-specific analyses led to activations in many foci, therefore suggesting a preferential involvement of these areas for these emotion conditions. As this aspect of the work is clearly exploratory with a threshold set to an uncorrected p = 0.001 and no *a priori* determined ROIs, results will need replication. Having said this, it is noteworthy that the hypothalamus was missing from the set of regions preferentially activated for the sexual arousal condition when contrasted with the other emotion conditions. Indeed, while it was clearly observed for the sexual arousal condition, it was not significantly more activated when compared with the other two emotion conditions. This somewhat contrasted with work from Walter et al. (2008) where hypothalamic activation was observed to be significantly stronger when viewing erotic pictures than when viewing non-erotic pictures matched for arousal, dominance, and valence. A possible explanation for this is the use, here, of film excerpts (that elicited hypothalamic activation for all three emotion conditions) in contrast to the use of still pictures as was done by Walter al. (2008). This said, it should be emphasized that the hypothalamus governs the endocrine system and is a major player in animal models of emotion in general (i.e. not just sexual arousal). It is involved in regulating motivated and homeostatic behavior and interacts with the autonomic nervous system through reciprocal connections with the PAG and other brainstem nuclei [8], [61], [62].

### Limitations and caveats

First, it is well known that emotions can influence respiration and heart rate. As these can in turn influence global BOLD signal, a concern could be that observed BOLD changes in the network identified here may essentially be due to respiration or heart rate-induced BOLD changes rather than to emotion-induced neuronal activation. This, however, is unlikely as respiration and heart rate effects have a characteristic spatial profile that spreads throughout gray matter with the largest signal changes being in the medial occipital cortex, precuneus, posterior cingulate, as well as in the vicinity of the posterior sagittal and transverse sinuses [63]. With the exception of a very small area within the right posterior cingulate gyrus (23 voxels), none of the above regions were observed in the conjunction analysis. Further, current findings were highly localized instead of widespread.

Second, current findings only pertain to the visual modality and to male subjects. Obviously, whether or not results would hold for female subjects or whether or not validated arousing emotion-inducing stimuli involving other sensory modalities would lead to overlapping regions of activation across emotional conditions remains to be ascertained.

Third, it could be argued that informing subjects, prior to scanning, that they would view film clips aimed at eliciting one of the three target emotions may have biased them towards these emotions. If such a bias was present, it would likely have led to less spatially extensive patterns of activation for each emotion condition as it would have increased the probability that the patterns of activation reflect, for a given condition, only contributions from the emotion under study rather than those from multiple emotions (under the assumption, of course, that multiple emotions each make a certain degree of specific contribution on top of the common regions of activation). As such, this potential bias could possibly have led to less extensive shared activation in the conjunction analysis. Assuming the existence of such a bias, conclusions regarding the large overlap between disparate emotional conditions would actually be strengthened as the overall pattern would be present despite a further increased probability of only eliciting the target emotion.

Fourth, testosterone levels are known to influence emotional behaviour (e.g. aggressive behaviour) [64] and to follow a circadian rhythm [65]. Further, testosterone has been, in previous work, positively associated with amygdalar and orbitofrontal reactivity during processing of emotional stimuli in healthy young [66] and middle-aged [67] men [64]. In light of this, it is possible that the exact time of scanning during the day may have influenced results. Here, all subjects were scanned in the afternoon; a time when testosterone tends to reach moderate levels. It could therefore be speculated that different trends of activation in the amygdala and prefrontal cortex could have been observed had scanning been done at other times during the day. Having said this, the amygdala and orbitofrontal cortex (which appear to be the main areas to be worried about in the current context) are among the brain areas most frequently observed in emotion studies (some of which were conducted 1) at presumably different hours than when our work was conducted and 2) on females subjects (i.e., in subjects having much lower testosterone levels). This suggests that modulation by testosterone likely had a small effect, if any, and therefore did not have a strong influence on the major conclusion of the study; namely that visual stimuli known to elicit various emotions evoke similar, large and distributed patterns of activation.

Finally, it is important to keep in mind that *f*MRI measures activity integrated across populations of neurons and that brain regions shown here to be consistently activated across emotional conditions are grossly defined, spanning at times a few cm in diameter. Such relatively large regions likely harbor various subsets of areas dedicated to different processes. In fact, areas spanning even a few mm are not consistently dedicated to any one process and individual neurons may participate in a number of functional circuits [8]. This suggests that finding a given region activated across different emotional conditions does not imply that a necessarily identical process is occurring within that region for different emotional states. Consistent with this view is the fact that in rats and humans, stimulation of brain sites that are only a few mm apart can elicit very different affective responses [68]. Nonetheless, the current results provide very clear evidence that viewing arousing/activating emotional stimuli leads to increases in activation that cluster within relatively confined and identifiable areas.

In summary, we have shown the existence of a general set of brain areas preferentially activated when male subjects view arousing validated film excerpts, regardless of their valence. Given that this network includes brain regions known from previous work to be intimately involved in homeostasis, arousal, appraisal, and attention, results could be speculated to suggest the existence of a set of areas meant to improve the way we deal with activating emotional stimuli as these are arguably the ones with the greatest potential of having an immediate impact on our lives. While this network is composed of most limbic areas, it further includes many areas not part of the limbic system. In light of this, results are only partially compatible with the limbic system theory and therefore support the view that it's obsolete [69].

## Supporting Information

Table S1 Voxel peak coordinates for the ‘amusement-neutral’ contrast.
(XLSX)

Table S2 Voxel peak coordinates for the ‘disgust-neutral’ contrast.
(XLSX)

Table S3 Voxel peak coordinates for the ‘sexual arousal-neutral’ contrast.
(XLSX)

Table S4 Voxel peak coordinates for the conjunction null analysis of the ‘amusement-neutral’, ‘disgust-neutral’, and ‘sexual arousal-neutral’ contrasts.
(XLSX)

Table S5 Regions more activated for the amusement than for the disgust and sexual arousal conditions.
(XLSX)

Table S6 Regions more activated for the disgust condition than for the amusement and sexual arousal conditions.
(XLSX)

Table S7 Regions significantly more activated for the sexual arousal condition than for the amusement and disgust conditions.
(XLSX)
